# The novel adipokine progranulin counteracts IL-1 and TLR4-driven inflammatory response in human and murine chondrocytes via TNFR1

**DOI:** 10.1038/srep20356

**Published:** 2016-02-08

**Authors:** Vanessa Abella, Morena Scotece, Javier Conde, Verónica López, Claudio Pirozzi, Jesús Pino, Rodolfo Gómez, Francisca Lago, Miguel Ángel González-Gay, Oreste Gualillo

**Affiliations:** 1 SERGAS (Servizo Galego de Saude) and IDIS (Instituto de Investigación Sanitaria de Santiago), Research Laboratory 9, The NEIRID Lab (Neuroendocrine Interactions in Rheumatology and Inflammatory Diseases), Santiago University Clinical Hospital, Santiago de Compostela, 15706, Spain; 2 Universidade da Coruña (UDC), Departamento de Bioloxía Celular e Molecular, Campus de A Coruña, A Coruña, 15071, Spain; 3 SERGAS (Servizo Galego de Saude), Division of Orthopaedics Surgery and Traumatology, Santiago University Clinical Hospital, Santiago de Compostela, 15706, Spain; 4 SERGAS (Servizo Galego de Saude) and IDIS (Instituto de Investigación Sanitaria de Santiago), Research Laboratory 7, Cellular and Molecular Cardiology Laboratory, Santiago University Clinical Hospital, Santiago de Compostela, 15706, Spain; 5 SERGAS (Servizo Galego de Saude), Division of Rheumatology, Santiago University Clinical Hospital, Santiago de Compostela, 15706, Spain; 6 Division of Rheumatology, Hospital Universitario Marqués de Valdecilla, IDIVAL, Santander, Cantabria, Spain

## Abstract

Progranulin (PGRN) is a recently identified adipokine that is supposed to have anti-inflammatory actions. The proinflammatory cytokine interleukin-1β (IL1β) stimulates several mediators of cartilage degradation. Toll like receptor-4 (TLR4) can bind to various damage-associated molecular patterns, leading to inflammatory condition. So far, no data exist of PGRN effects in inflammatory conditions induced by IL1β or lipopolysaccharide (LPS). Here, we investigated the anti-inflammatory potential of PGRN in IL1β- or LPS-induced inflammatory responses of chondrocytes. Human osteoarthritic chondrocytes and ATDC-5 cells were treated with PGRN in presence or not of IL1β or LPS. First, we showed that recombinant PGRN had no effects on cell viability. We present evidence that PGRN expression was increased during the differentiation of ATDC-5 cell line. Moreover, PGRN mRNA and protein expression is increased in cartilage, synovial and infrapatellar fat pad tissue samples from OA patients. PGRN mRNA levels are upregulated under TNFα and IL1β stimulation. Our data showed that PGRN is able to significantly counteract the IL1β-induced expression of NOS2, COX2, MMP13 and VCAM-1. LPS-induced expression of NOS2 is also decreased by PGRN. These effects are mediated, at least in part, through TNFR1. Taken together, our results suggest that PGRN has a clear anti-inflammatory function.

Osteoarthritis (OA) is a multifactorial joint degenerative disease characterized by progressive destruction of articular cartilage, changes in subchondral bone, osteophyte formation and synovial inflammation. It is the most prevalent type of arthritis, but its aetiology is still largely unknown^1^. Although OA is commonly described as non-inflammatory disease, inflammation is recognized as contributing to the symptoms and progression of OA^2^. On the other side, obesity is an important risk factor for OA that may result in overloading of joints and by a chronic ‘low-grade inflammatory systemic state sustained by a dysregulation of adipokines in white adipose tissue and other peripheral tissues, including joint tissues, that can contribute to an altered immune and inflammatory response^3^,^4^. Several adipokines can also be produced by chondrocytes and act locally in cartilage homeostasis^5^,^6^.

Progranulin (PGRN) is a recently identified adipokine^7^, also known as GP88, acrogranin, proepithelin, granulin/epithelin precursor (GEP) or PC cell-derived growth factor (PCDGF). PGRN is a 68–88 kDa secreted glycoprotein that is produced by a broad range of tissues, including human articular cartilage^8^ and adipose tissue^7^. PGRN has been implicated in a wide variety of biological functions, including wound healing^9^, bone regeneration^10^, and inflammation^11^,^12^. It has been previously reported that PGRN levels were significantly elevated in cartilage of patients with OA and rheumatoid arthritis (RA)^8^. Moreover, serum levels of PGRN in RA patients were found to be significantly higher than those in age-matched healthy controls^13^. Recently, it has been demonstrated that PGRN levels are increased at local site of inflammation and are associated to disease activity in patients with RA^14^. PGRN also plays a crucial role in chondrocyte proliferation^15^, differentiation and endochondral ossification of growth plate during development^16^,^17^. In addition, the group of Chuanju Liu reported that PGRN antagonised tumour necrosis factor α (TNF-α) through binding to TNF receptors (TNFR), and exhibited an anti-inflammatory function, by suppressing the pro-inflammatory action of TNF-α in a arthritis murine models^18^,^19^. Additionally, these authors found that deficiency of PGRN led to spontaneous OA-like phenotype in ‘aged’ mice. PGRN-deficient mice exhibited breakdown of cartilage structure, while local delivery of recombinant PGRN protein attenuated degradation of cartilage matrix in surgically induced OA models. Furthermore, PGRN plays a protective role by promoting anabolism of degenerative chondrocytes mainly through TNF receptor 2 (TNFR2). TNF receptor 1 (TNFR1) binding to PGRN prevents the activation of the NF-κB pathway by TNFα, which induces MMPs and ADAMTS, thus inhibiting cartilage degradation^20^.

Secreted pro-inflammatory cytokines, such as Interleukin 1β (IL1β), are critical mediators implicated in OA pathophysiology, which make them primary targets for therapeutic strategies^21^. IL1β is released by synoviocytes, chondrocytes, and invading macrophages in inflamed joints and it is well-established that IL1β plays a pivotal role in the pathogenesis of OA^22^. IL1β triggers a cascade of cartilage damage events like the production of more pro-inflammatory cytokines, the synthesis of catabolic factors and the release of some inflammatory mediators such as prostaglandins produced by COX-2 and nitric oxide (NO) up-regulated by NOS2^23^. Moreover, it has been suggested that IL1β induces the expression of the TNF-α gene in chondrocytes^24^ and upregulates the surface expression of TNFRs^25^.

Given that innate immune responses are important in the development of OA, the role of Toll like receptors (TLR) have been recently taken into account^26^. Among TLRs, TLR4 has been involved in OA^27^. TLR4 activation by lipopolysaccharide (LPS) involves the production of NO^28^, several pro-inflammatory cytokines^29^ and adipokines^5^,^30^ that may work together boosting cartilage degradation^31^. Previously, Yin *et al.* reported that PGRN-deficient macrophages challenged with LPS increase pro-inflammatory cytokine production^32^, and Hwang *et al.* showed that PGRN efficiently inhibited LPS-mediated pro-inflammatory signalling in endothelial cells through attenuation of the NF-κB pathway, suggesting its beneficial anti-inflammatory effects^33^.

To the best of our knowledge, no data exist about PGRN effect on IL1β- or LPS-induced inflammatory responses of chondrocytes. Therefore, we investigated the effect of PGRN on the expression of some inflammatory mediators and catabolic factors in IL1β- or LPS-stimulated chondrocytes for a better understanding of the underlying mechanisms implicated.

## Materials and Methods

For experiments involving humans, all the methods were carried out in accordance with the approved guidelines. All experimental protocols were approved by the local ethics committee (SERGAS, Santiago University Clinical Hospital Ethics Committee (CAEIG Comité Autonómico de Ética da Investigación de Galicia 2014/310). Informed consent was obtained from all subjects.

### Reagents

Fetal bovine serum (FBS), MTT dye, human transferrin, sodium selenite, ethylenediaminetetraacetic acid (EDTA) solution, mouse recombinant TNF-α, mouse and human recombinant IL1β and lipopolysaccharide (LPS) were obtained from Sigma (St. Louis MO, USA). Dulbecco’s modified Eagle’s medium (DMEM)/Ham’s F12 medium, l-glutamine, antibiotics and trypsin-EDTA were purchased from Lonza (Verviers, Belgium). Recombinant mouse PGRN (18–589) was purchased from Enzo Life Sciences (NYC, USA) and recombinant human PGRN (1–593) was from AdipoGen (Switzerland).

### Cell culture and treatments

The murine chondrogenic cell line ATDC-5 (purchased from RIKEN Cell Bank, Tsukuba, Japan) was cultured in DMEM–Ham’s F-12 medium supplemented with 5% fetal bovine serum, 10 μg/ml human transferrin, 3·10^−8^ M sodium selenite, l-glutamine, and antibiotics (50 units/ml penicillin and 50 μg/ml streptomycin). Chondrogenic ATDC-5 cells were differentiated into mature chondrocytes. Briefly, cells were seeded at a density of 6·10^4^/well in 6-well plates with the ATDC-5 standard media supplemented with insulin (10 mg/mL). The differentiation media was replaced every two days for 14 days. Differentiation was qualitatively characterized by increased formation of cell nodules. In other experiments (data not shown), differentiation was further analyzed by sequential increase in the levels of type II collagen, aggrecan and type X collagen mRNA, as previously published^34^. The immortalized human juvenile costal chondrocyte cell line T/C-28a2 (a kind gift from Dr. M.B. Goldring, Hospital for Special Surgery, NYC, USA) was culture in DMEM–Ham’s F-12 medium supplemented with 10% fetal bovine serum, l-glutamine, and antibiotics (50 units/ml penicillin and 50 μg/ml streptomycin). Primary chondrocytes were harvested from human OA articular cartilage samples obtained from articular joints of patients undergoing total knee replacement surgery (with permission from the local ethics committee (SERGAS, Santiago University Clinical Hospital Ethics Committee (CAEIG Comité Autonómico de Ética da Investigación de Galicia 2014/310) and informed consent was obtained from all patients participating in the study) as previously described^35^. Human chondrocytes were cultured in DMEM/Ham’s F12 medium supplemented with 10% of fetal bovine serum, L-glutamine, and antibiotics (50 units/ml penicillin and 50 mg/ml streptomycin). Cells were seeded in monolayer up to the high density and used in the first passage of culture in order to avoid dedifferentiation.

For RT-PCR and western blot, cells were seeded in 6-well plates until complete adhesion and then incubated overnight in serum-free conditions. Then, cells were treated as indicated in each case.

### Cell viability

Cell viability was tested using the methyl-thiazolyl-tetrazolium (MTT) reagent to detect functional mitochondria in living cells. Briefly, ATDC-5, ATDC-5 mature and T/C-28a2 cells (8·10^3^/well) were seeded in 96-well plates. After overnight starvation, they were incubated with recombinant mouse and human PGRN for 48 h in serum free medium. Doses of 100 and 200 ng/ml of PGRN reflect serum concentrations in healthy individuals^36^,^37^. Then, cells were incubated for 4 h with MTT reagent. After formazan salt was dissolved, absorbance was measured at 550 nm using a microtiter enzyme-linked immunosorbent assay reader (Multiskan EX; Thermo Labsystems). Cells cultured without PGRN were used to normalize cell viability status.

### Nitrite assay

Nitrite accumulation was determined in the culture medium using the Griess reaction, as previously described^31^. Briefly, 100 μl cell culture medium was mixed with 100 μl Griess reagent (equal volumes of 1% [weight/vol] sulfanilamide in 5% [vol/vol] phosphoric acid and 0.1% [weight/vol] naphtylethylenediamine-HCl), incubated at room temperature for 10 min, and then the absorbance at 550 nm was measured using a microplate reader (Titertek-Multiscan, Labsystem, Helsinki, Finland). Fresh culture medium was used as blank in all of the experiments. The amount of nitrite in the samples (in micromolar units) was calculated from a sodium nitrite standard curve freshly prepared in culture medium.

### RNA Isolation and Real-time Reverse Transcription-Polymerase Chain Reaction (RT-PCR)

Mouse and human PGRN mRNA levels were determined using SYBR Green–based quantitative PCR. RNA was extracted using a NucleoSpin kit (Macherey-Nagel), according to the manufacturer’s instructions. For relative quantification, we performed an RT reaction using 1 mg of RNA with a Thermo Scientific Verso cDNA Synthesis Kit (42 °C for 30 min, followed by incubation at 95 °C for 2 min). RT-PCR was performed in a Stratagene MX3005P thermal cycler using a standard protocol (95 °C for 10 min followed by 40 cycles for 15 s of denaturation at 95 °C and 1 min annealing/extension at 60 °C), a SABiosciences Master Mix and specific primers (for mouse GAPDH, 140 bp, PPM02946E, reference position 309, GenBank accession no. NM_008084.2; for human GAPDH, 175 bp, PPH00150E, reference position 1287, GenBank accession no. NM_002046.3; for mouse PGRN, 121 bp, PPM24687A, reference position 1704, GenBank accession no. NM_008175.4; for human PGRN, 130 bp, PPH02482B, reference position 1975, GenBank accession no. NM_002087.2; for mouse NOS2, 122 bp, PPM02928B, reference position 2728–2748, GenBank accession no. NM_010927.3; for mouse TNFRSF1A, 156 bp, PPM03087D, reference position 1461, GenBank accession no. NM_ 011609.4; for mouse TNFα, 110 bp, PPM03113G, reference position 749, GenBank accession no. NM_013693). Results of comparative RT-PCRs were analyzed using MxPro software (Stratagene, CA, USA).

### Protein extraction and Western blot analysis

After the cell treatment, cells were rapidly washed with ice-cold phosphate buffered saline and scraped in lysis buffer for protein extraction (10 mM Tris/HCl, pH 7.5, 5 mM EDTA, 150 mM NaCl, 30 mM Sodium pyrophosphate, 50 mM sodium fluoride, 1 mM sodium orthovanadate, 0.5% Triton X-100, 1 mM PMSF and protease inhibitor cocktail from Thermo Scientific). Lysed cells were centrifuged at 14.000 g for 20 min. SDS-PAGE and blotting procedure were carried on as previously described^38^. Immunoblots were incubated with the appropriate antibody (anti-PGRN diluted 1:1000, Santa Cruz, CA, USA; anti-NOS2 diluted 1:1000, Cell Signalling, MA, USA; anti-COX2 diluted 1:50, anti-VCAM-1 diluted 1:1000, Cell Signalling, MA, USA; anti-MMP13 diluted 1:500, Santa Cruz, CA, USA) and visualized with an Immobilon Western Detection kit (Millipore, MA) using anti-rabbit (GE Healthcare, UK) horseradish-peroxidise-labelled secondary antibody diluted 1:2000. To confirm equal loading in each sample, the membranes were stripped in stripping buffer (100 mM β-mercaptoethanol, 2% SDS, 62.5 mM Tris-HCl pH 6.7) and re-blotted with anti-GAPDH antibody diluted 1:30000 (Sigma, MO, USA). The images were captured and analyzed with an EC3 imaging system (UVP). Densitrometric analyses were performed using ImageJ software (National Institutes of Health, Bethesda, MD, USA).

### siRNA-mediated gene silencing of TNFα receptor

In order to silence TNFR gene, we used the siRNA that targets TNFRSF1A (Integrated DNA Technologies, USA) and the siRNA negative control, that does not target any known sequence. Transfection with 10 nM of siRNA duplex was performed using the cationic lipid siLentFect (BioRad, CA, USA) according to the manufacturer’s recommendations. Cells were transfected for 48 h and then they were stimulated with 0.1 ng/ml of IL1β in presence or not of 200 ng/ml of PGRN for 48 h.

### Statistical analysis

Data are reported as the mean ± standard error of the mean (SEM) and followed normal distribution of independent experiments, which were done at least three times, each time with at least three independent observations. Statistical analysis was performed using two-tailed Student’s t test and one-way ANOVA test followed by the Bonferroni’s test for multiple-comparisons using the Prism computerized package (GraphPad Software V.5, La Jolla, CA, USA). P-values less than 0.05 were considered significant.

### Ethics approval

This study was conducted with the approval of the Santiago University Clinical Hospital Ethics Committee (CAEIG 2014/310).

## Results

### Effect of PGRN on cell viability

First, in order to assess the effect on vitality of PGRN, we performed MTT assay in undifferentiated ATDC-5, in mature ATDC-5 and human T/C-28a2 chondrocytes. Treatment with increasing concentrations of PGRN for 48 hours showed no toxic effect on cell vitality (Sup. Fig. 1).

### PGRN mRNA and protein production during ATDC-5 differentiation

**Figure 1:**
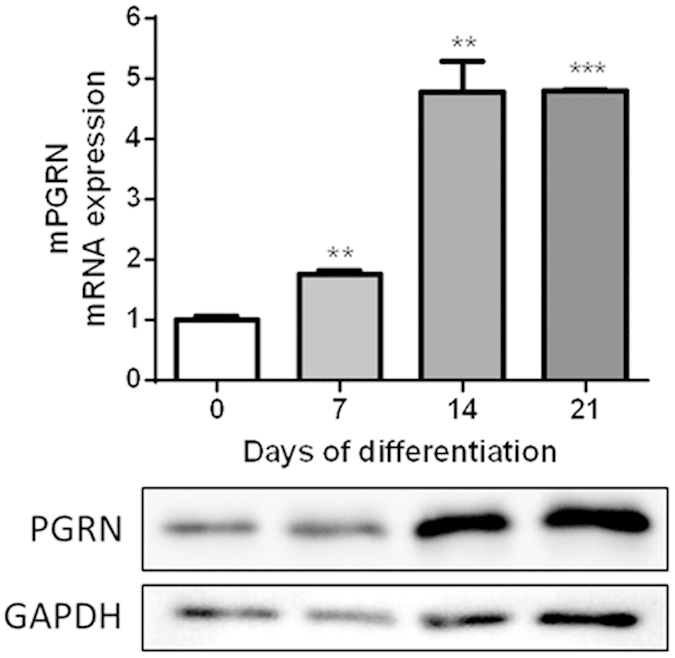
PGRN mRNA and protein expression during ATDC-5 differentiation after 7, 14, and 21 days. Values are the mean ± SEM of at least 4 independent experiments (**P < 0.01 *vs.* Ctrl, ***P < 0.001 *vs.* Ctrl.). Cell lysates underwent Western blot analysis using PGRN antibody. GAPDH was used as a loading control. The blots were run under the same experimental conditions. The full blots are shown in Supplementary Fig. 2. Blots are representative of at least 3 independent experiments.

To determine whether the levels of PGRN mRNA and protein change during chondrocyte differentiation, we differentiated ATDC-5 cells into mature and hypertrophic chondrocytes. As shown in Fig. 1, PGRN mRNA expression increased along differentiation process of ATDC-5 cells. This increase is highly significant after 7, 14, and 21 days of differentiation in comparison to undifferentiated cells (Day 0) (Fig. 1, upper panel). This effect was observed also at protein expression (Fig. 1, lower panel).

### PGRN expression in osteoarthritic and healthy tissues

**Figure 2:**
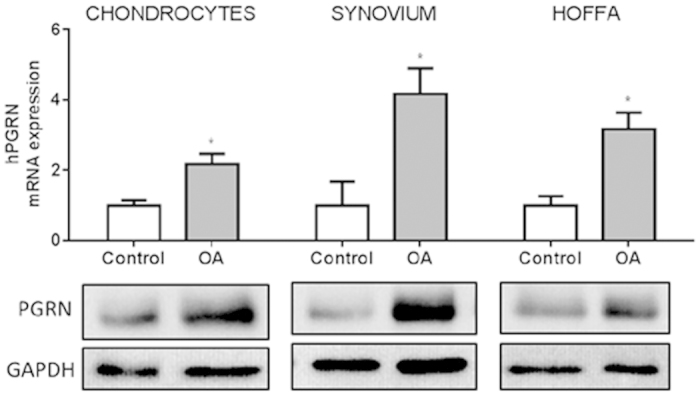
Determination of PGRN mRNA and protein in healthy and osteoarthritis human tissues. PCR results were shown in fold change, where grey bars represent the PGRN mRNA expression in chondrocytes, infrapatellar fat pads (IPFPs) and synovial tissues obtained from osteoarthritis (OA) patients (*P < 0.05 *vs.* Ctrl.). PGRN protein expression was showed by representative western blots of 18 OA patients (age 52–73; mean BMI 28.4) and 6 healthy donors (age 23–50; mean BMI 23.4). GAPDH was used as loading control. Western blots have been run under the same experimental conditions. The full blots are shown in Supplementary Fig. 3.

Since local production of adipokines may have important pathological implications on cartilage homeostasis, we also assessed the expression of PGRN mRNA and protein levels in human primary chondrocytes, infrapatellar fat pad (IPFP) and synovium of OA patients, and compare this expression between OA subjects and healthy donors. As shown in Fig. 2, human primary chondrocytes, IPFP and synovium expressed efficiently PGRN. Moreover, PGRN expression was significantly higher in chondrocytes, IPFP and synovial tissues obtained from OA patients compared to those obtained from healthy donors.

### Effect of pro-inflammatory cytokines and TLR-4 agonist on PGRN mRNA expression in ATDC5 differentiated cells

**Figure 3:**
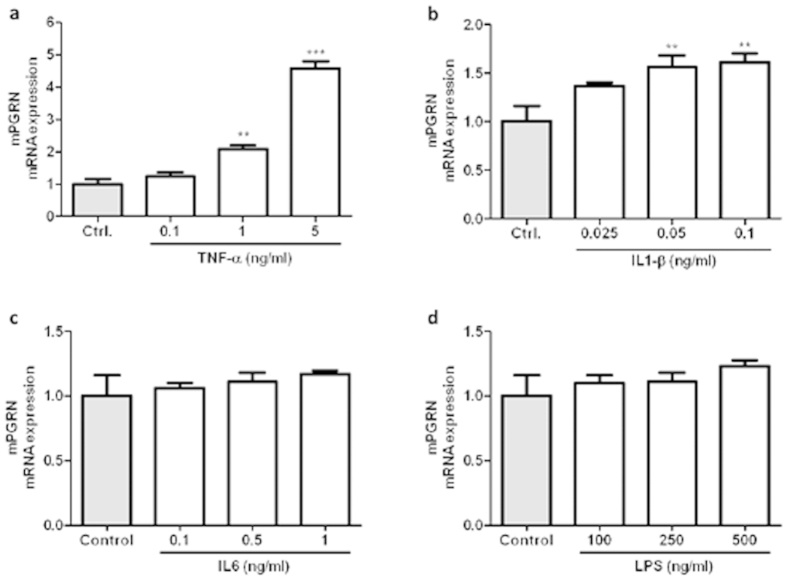
Mouse PGRN mRNA expression after TNFα, IL1β, IL6 and LPS treatment. (**a**) Cells were treated with TNFα 0.1, 1, 5 ng/ml, (**b**) IL1β 0.025, 0.05, 0.1 ng/ml, (**c**) IL6 0.1, 0.5, 1 ng/ml and (**d**) LPS 100, 250, 500 ng/ml for 24 h. Values are the mean ± SEM of at least 3 independent experiments (**P < 0.01 *vs.* Ctrl, ***P < 0.001 *vs.* Ctrl.).

To elucidate the pattern of PGRN expression under pro-inflammatory conditions, we treated differentiated ATDC-5 (14 days) cells with different doses of TNFα, IL1β, IL6 and LPS as a TLR-4 agonist. These differentiated cells have a phenotype of adult mature chondrocytes and, in spite of its murine origin, have an identical behaviour to human cells, at last in terms of inflammatory mediators expression^39^. As shown in Fig. 3a, cells stimulated with TNFα during 24 h showed a marked increase in PGRN mRNA expression at 1 and 5 ng/ml. A similar effect was observed when cells were stimulated with IL1β (0.05 and 0.1 ng/ml; Fig. 3b) during 24 h. Nonetheless, neither IL6 nor LPS had significant effects on PGRN expression (Fig. 3c,d).

### PGRN decreased IL1β induction of chondrocyte catabolism

**Figure 4:**
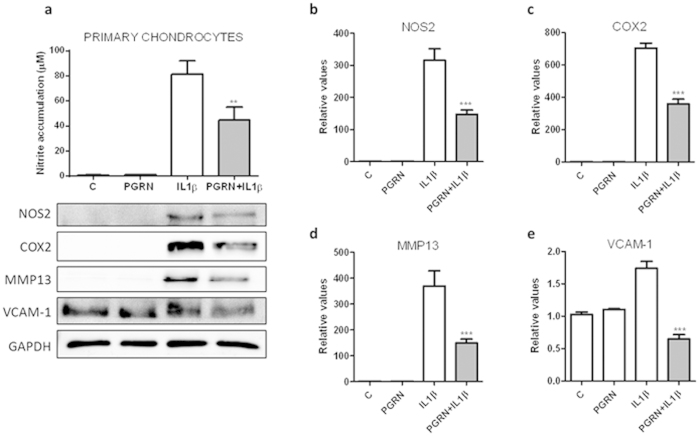
PGRN suppressed IL1-β induction of chondrocyte catabolism. (**a**) Cells were treated with 10 ng/ml of IL1β, in presence or absence of 200 ng/ml of PGRN for 48 h. Culture medium was subsequently analyzed for nitrite levels. NO concentration (μM) was determined using the Griess reaction. Values are the mean ± SEM of at least 3 independent experiments (**P < 0.01 *vs.* IL1β) (**a**, upper panel). Cell lysates underwent Western blotting analysis under the same condition (**a**, lower panel). After transferring the blots onto PVDF membranes, we cropped the targeted blots according to referenced indicating markers, and then targeted proteins were immunoblotted with NOS2, COX-2, MMP13 and VCAM-1 antibodies. GAPDH was used as a loading control. Blots are representative of at least 3 independent experiments. (**b–e**) Western blot densitometric analysis (n = 3; ***P < 0.001 PGRN + IL1β *vs.* IL1β).

In order to gain further insights into the potential pharmacological activity of PGRN, human primary chondrocytes were treated with IL1β (10 ng/ml) in presence or not of PGRN 200 ng/ml for 48 h. As shown in Fig. 4a, human primary chondrocytes challenged with IL1β, showed a strong accumulation of NO after 48 h in comparison to control unstimulated cells. However, treatment with recombinant PGRN significantly (P < 0.01) led to inhibition of IL1β-induced NO production, whereas PGRN per se did not affect basal NO production. Nitrite accumulation inhibition was further confirmed by western blot analysis of NOS type 2 (NOS2). PGRN was able to clearly inhibit NOS2 protein expression (Fig. 4b). Furthermore, since PGRN is supposed to prevent cartilage destruction in inflammatory arthritis mediated by TNF-α, we examined the levels of catabolic biomarkers COX-2 and MMP13 in human primary chondrocytes challenged with IL1β. COX-2 and MMP13 protein levels were significantly increased after 48 h of IL1β treatment. However, PGRN significantly decreased IL1β-induced COX-2 and MMP13 expression (Fig. 4c,d). Moreover, since pro-inflammatory cytokines such as IL1β and TNFα are able to up-regulate VCAM-1 expression in primary cultures of human articular chondrocytes, we examined VCAM-1 protein levels after PGRN treatment. IL1β is able to induce VCAM-1 expression. The treatment with PGRN produced a significant decrease of IL1β-induced VCAM-1 expression (Fig. 4e).

### PGRN counteracted IL1β and LPS-driven NOS2 through TNFR1

**Figure 5:**
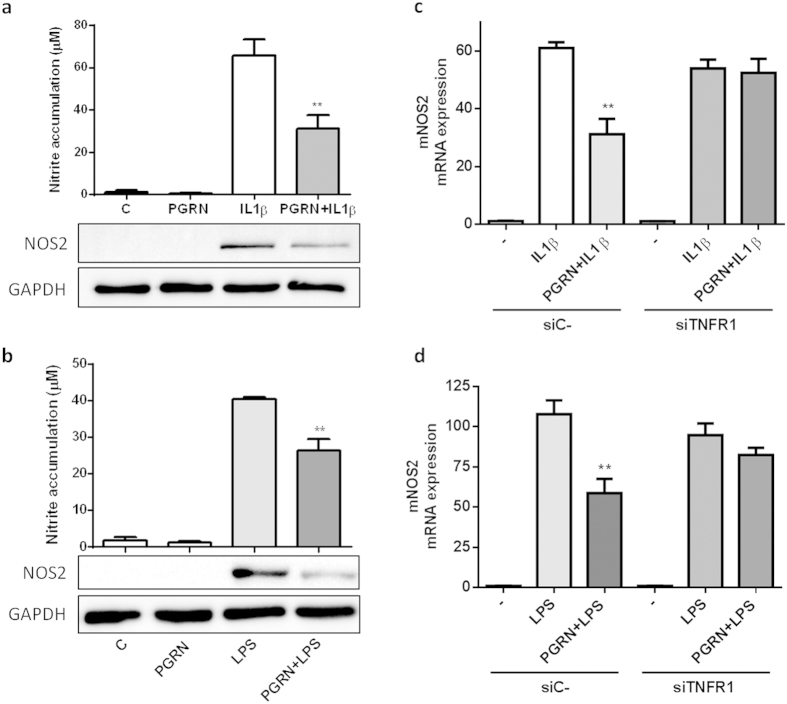
PGRN counteracted IL1β and LPS-induced nitric oxide (NO) production and inhibited NOS2 expression in cultured ATDC-5 cells, at least in part, through TNFR1. (**a,b**) Cells were treated with 0.1 ng/ml of IL1β (**a**) and 250 ng/ml of LPS (**b**) alone or in combination PGRN (200 ng/ml) during 48 h. Culture medium was subsequently analyzed for nitrite levels. NO concentration (μM) was determined using the Griess reaction. Values are the mean ± SEM of at least 3 independent experiments (**a**) **P < 0.01 PGRN + IL1β *vs.* IL1β; (**b**) **P < 0.01 PGRN + LPS *vs.* LPS). Cell lysates underwent Western blotting analysis using NOS2 antibody. GAPDH was used as a loading control. The blots were run under the same experimental conditions. The full blots are shown in Supplementary Fig. 3. Blots are representative of at least 3 independent experiments. (**c,d**) Cells were transfected with negative control siRNA or TNFR1-targeted siRNA (siTNFR1) and stimulated with 0.1 ng/ml of IL1β (**c**) and 250 ng/ml of LPS (**d**) in absence or presence of PGRN 200 ng/ml for 48 h. Relative mRNA levels of NOS2 were measure by RT-PCR. Values are the mean ± SEM of at least 3 independent experiments (**c**) **P < 0.01 siC- PGRN + IL1β *vs.* siC- IL1β; (**d**) **P < 0.01 siC- PGRN + LPS *vs.* siC-LPS).

It was reported that PGRN can competitively bind to TNFR1 and TNFR2 and prevents TNFα-mediated inflammation^18^. On the basis of above introduced results showing that PGRN counteracts IL1β-driven inflammatory response, we sought to investigate whether this anti-inflammatory effect of PGRN was mediated through TNFR. Firstly, we corroborated that PGRN was able to modulate IL1β or LPS-induced NO levels and NOS2 expression also in differentiated ATDC5 cells (Fig. 5a,b). Then, to determine whether the anti-inflammatory effect of PGRN was mediated through TNFR, we analysed the role of TNFR1 using a siRNA approach. Mature ATDC5 cells were transfected with siTNFR1 and then treated with IL1β or LPS in presence or not of PGRN (Fig. 5c,d). As shown in Fig. 5, TNFR1 siRNA knockdown supressed the anti-inflammatory effect of PGRN under IL1β (panel c) or LPS (panel d) stimulation.

## Discussion

The recently discovered adipokine PGRN has been matter of extensive studies in the last few years. The role of PGRN in inflammation has been only partially defined^40^. Although PGRN is one of the major anti-inflammatory molecules, it is possible that PGRN has dual roles in inflammation and might exert pro- or anti-inflammatory functions depending on different tissues. For instance, PGRN inhibits LPS-mediated IL-6, TNF-α, and MCP-1 cytokine release from macrophages^41^, but PGRN is also the major adipokine involved in high-fat diet-induced insulin resistance by inducing up-regulation of IL-6 expression, a pro-inflammatory cytokine^7^. Chronic inflammation in RA is mainly mediated by TNF-α^42^. Recently, PGRN was recognized as a novel ligand of TNFR1/2. Actually, PGRN blocks TNF-α-mediated signalling pathways by competing with TNF-α binding to receptors in inflammatory arthritis murine models^18^,^19^.

However, there are currently no approaches in the literature evaluating PGRN action neither through IL1β nor through TLR4 activation in chondrocytes. Previously, Yin *et al.* reported that PGRN-deficient macrophages challenged with LPS increase pro-inflammatory cytokine production^32^, and Hwang *et al.* showed that PGRN efficiently inhibited LPS-mediated pro-inflammatory signalling in endothelial cells through attenuation of the NF-κB pathway, suggesting its beneficial anti-inflammatory effects^33^. Thus, we have studied the effect of PGRN on the expression of some inflammatory mediators and catabolic factors in IL1β- or LPS-stimulated chondrocytes. To the best of our knowledge, this is the first experimental evidence that PGRN is able to reduce mediators of inflammation induced by IL1β or LPS in human chondrocytes.

Here we show for the first time, that PGRN mRNA and protein expression is detected in human infrapatellar fat pads (IPFPs) and synovial tissues. Moreover, PGRN expression was significantly higher in chondrocytes, IPFP and synovial tissues obtained from OA patients compared to these tissues obtained from healthy donors. Our findings are in agreement with previous results of increased PGRN mRNA and protein expression in OA and RA cartilage described by Guo *et al.*^8^ Recently, it was reported the expression profile of PGRN in another cartilage degenerative disease, such as disc degenetation. PGRN levels were also elevated in intervertebral discs during aging, and PGRN knockout mice developed an early onset of degeneration in intervertebral cartilage^43^.

Our work makes another novel observation, that PGRN mRNA expression was increased during the process of ATDC-5 cell differentiation into mature chondrocytes. These data suggested that this adipokine might play a role in the complex mechanisms of chondrocyte development and differentiation. PGRN might be a marker of late-phase chondrogenic differentiation. To this regard, the group of Chuan-ju Liu reported that PGRN was a key downstream molecule of BMP2, and it was required for BMP2-mediated chondrocyte differentiation through Erk1/2 signaling^17^. They also showed that PGRN was required for BMP-2 induction of osteoblastogenesis and ectopic bone formation^10^. Moreover, we observed elevated PGRN levels also in hypertrophic chondrocytes, those localized above subchondral bone. However, in PGRN −/− mice, Zhao *et al.* revealed osteophyte formation and ectopic subchondal sclerosis. These results indicated disorder of bone metabolism in cartilage and subchondral bone of these mice, suggesting a role for PGRN in the regulation of subchondral bone turnover^20^.

Although several suppositions postulated that PGRN might be strongly influenced by the inflammatory “milieu”, here we show clear experimental evidence that classic cytokines or activation of TLR4 by LPS in chondrocytes are able to strongly influence PGRN levels. These findings suggest that PGRN induction by TNFα and IL1β released from joint tissues may be an adaptive response to the inflammatory state.

Since IL1β plays a pivotal role in the pathogenesis of OA^22^, counteracting its pro-inflammatory effects with PGRN might be an interesting therapeutic strategy. Actually, we assessed whether PGRN was able to inhibit IL1β-mediated pro-inflammatory response. Our data clearly demonstrate that PGRN strongly attenuated IL1β driven inflammatory markers, such as NO production, NOS2, COX2 and VCAM-1 expression, as well as catabolic markers of cartilage breakdown, such as MMP13. Moreover, we showed clear evidence that PGRN effect upon IL1β-driven NOS2 induction is mediated, at least in part, through TNFR1. Actually, by down-regulating TNFR1 expression through RNA interference, the anti-inflammatory role of PGRN counteracting IL1β was totally blocked, suggesting that PGRN is working via TNFα signalling through binding to TNFR.

Here we also show clear evidence that PGRN is able also to modulate LPS-driven NOS2 induction. This effect is likely to be mediated, at least in part, through TNFR1. Actually, by down-regulating TNFR1 expression through siRNA, the anti-inflammatory role of PGRN was totally blocked. Our results are in line with other published results suggesting that PGRN is able to inhibit LPS-mediated pro-inflammatory signalling^32^,^33^. Thus, at the functional aspect, the activity of PGRN, and its increased expression observed upon IL-1β and TNF-alpha treatment could mediate and sustain clear pro-anabolic and anti-inflammatory pathways. Nonetheless, the relevance of these data in the pathophysiology of osteoarthritis or other chronic rheumatic diseases should be supported by clinical data that at present are non-existent.

In conclusion, on the basis of our data and on previous published results, PGRN in articular joint cells could limit expression of inflammatory mediators, adhesion molecules and enzymes involved in cartilage breakdown triggered by cytokines and LPS that ultimately compromise cell survival and tissue function. PGRN may represent a novel key molecule coupling cartilage metabolism with inflammation to switch physiologic and clinical phenotypes. Thus, targeted strategies aimed to increase PGRN levels appeals to be an attractive therapeutic strategy for OA, which could be potentially achieved through multiple ways including life style modifications and pharmacological approaches.

## Additional Information

**How to cite this article**: Abella, V. *et al.* The novel adipokine progranulin counteracts IL-1 and TLR4-driven inflammatory response in human and murine chondrocytes via TNFR1. *Sci. Rep.* **6**, 20356; doi: 10.1038/srep20356 (2016).

## Supplementary Material

Supplementary Information
