# Supplementary material for: The novel adipokine progranulin counteracts IL-1 and TLR4-driven inflammatory response in human and murine chondrocytes via TNFR1
Source: Sci Rep. 2016 Feb 8;6:20356. doi: 10.1038/srep20356 (PMC4745010; doi:10.1038/srep20356)

**Supplementary Information for Abella et al.**

**The novel adipokine progranulin counteracts IL-1 and TLR4-driven inflammatory response in human and murine chondrocytes via TNFR1**

Vanessa Abella^1-2^, Morena Scotece^1^, Javier Conde^1^, Verónica López^1^, Claudio Pirozzi^1^, Jesús Pino^3^, Rodolfo Gómez^1^, Francisca Lago^4^, Miguel Ángel González-Gay^5^ and Oreste Gualillo^1*^

^1^ SERGAS (Servizo Galego de Saude) and IDIS (Instituto de Investigación Sanitaria de Santiago), Research Laboratory 9, The NEIRID Lab (Neuroendocrine Interactions in Rheumatology and Inflammatory Diseases), Santiago University Clinical Hospital, Santiago de Compostela, 15706, Spain.

^2^ Universidade da Coruña (UDC), Departamento de Bioloxía Celular e Molecular, Campus de A Coruña, A Coruña, 15071, Spain.

^3^ SERGAS (Servizo Galego de Saude), Division of Orthopaedics Surgery and Traumatology, Santiago University Clinical Hospital, Santiago de Compostela, 15706, Spain.

^4^ SERGAS (Servizo Galego de Saude) and IDIS (Instituto de Investigación Sanitaria de Santiago), Research Laboratory 7, Cellular and Molecular Cardiology Laboratory, Santiago University Clinical Hospital, Santiago de Compostela, 15706, Spain.

^5^SERGAS (Servizo Galego de Saude), Division of Rheumatology, Santiago University Clinical Hospital, Santiago de Compostela, 15706, Spain.

***Correspondence to: Dr. Oreste Gualillo,** email: [oreste.gualillo@sergas.es](mailto:oreste.gualillo@sergas.es)

**Supplementary Figure Legends**

**Supplementary Figure 1.** **Effect of PGRN on cell viability.** (**a**) Undifferentiated ATDC-5 chondrocytes, (**b**) mature ATDC-5 cells and (**c**) human T/C-28a2 cells were incubated with PGRN (100, 200, 500 ng/ml) for 48 h and analyzed using a MTT colorimetric assay. Results represent mean ± SEM of at least 3 independent experiments, each with at least 8 independent observations.

**Supplementary Figure 2.** Supplementary full scans of Western blots of PGRN and GAPDH in Figure 1. Full scans were cropped and used in the indicated primary figure.

**Supplementary Figure 3.** Supplementary full scans of Western blots of PGRN and GAPDH in Figure 2. Full scans were cropped and used in the indicated primary figures.

**Supplementary Figure 4.** Supplementary full scans of Western blots of NOS2 and GAPDH in Figure 5a and b. Full scans were cropped and used in the indicated primary figures.

**Supplementary Figures**

**Supplementary Figure 1.**


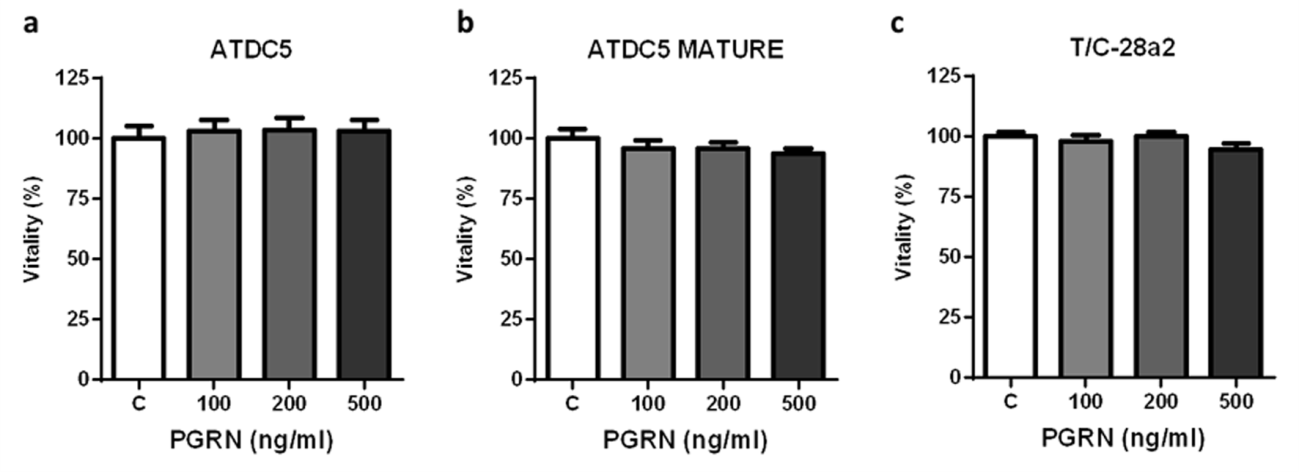


**Supplementary Figure 2.**

**
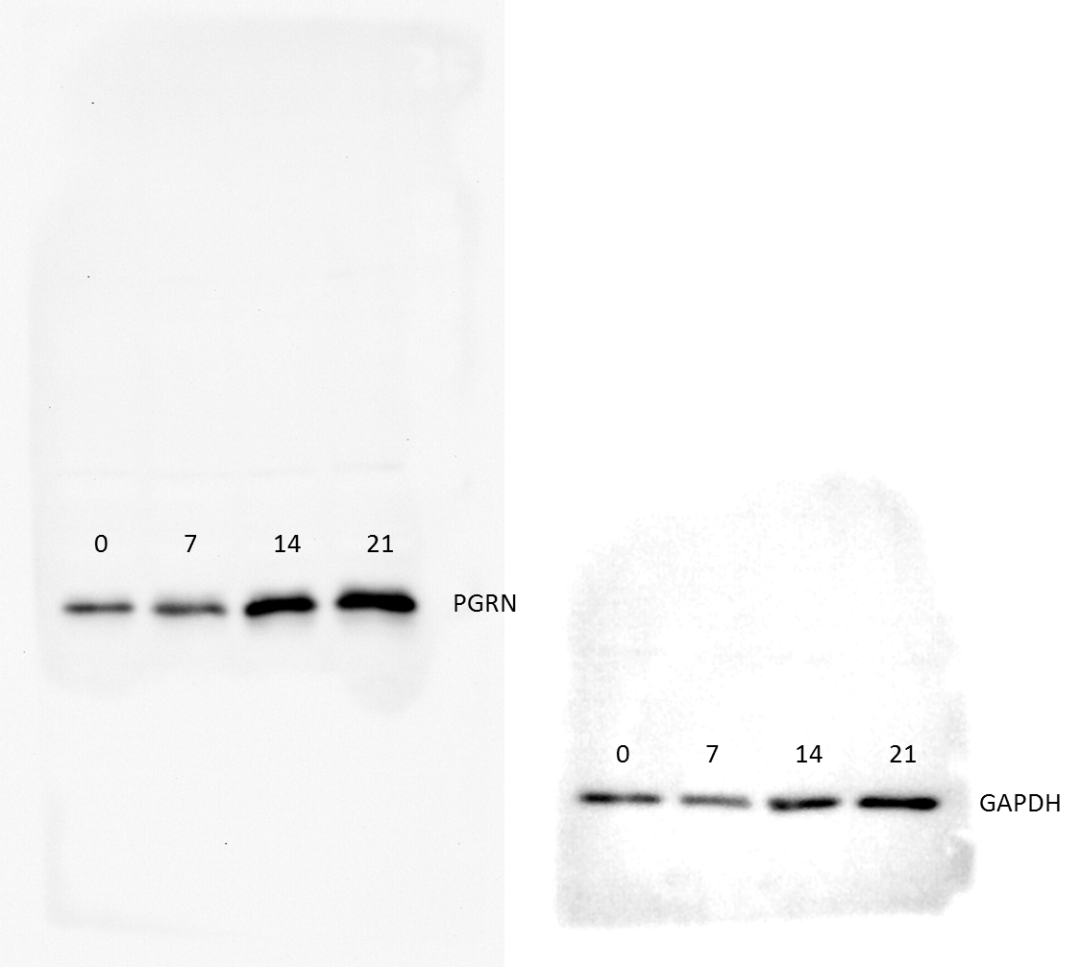
**

**Supplementary Figure 3.**

**
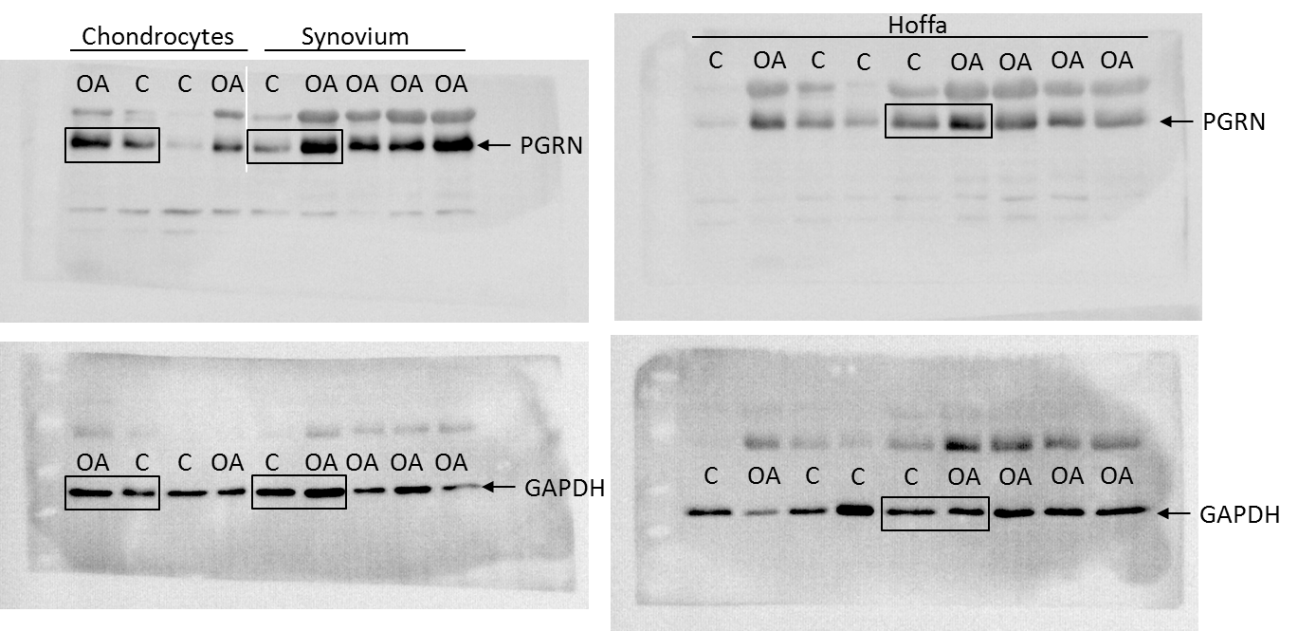
**

**Supplementary Figure 4.**

**
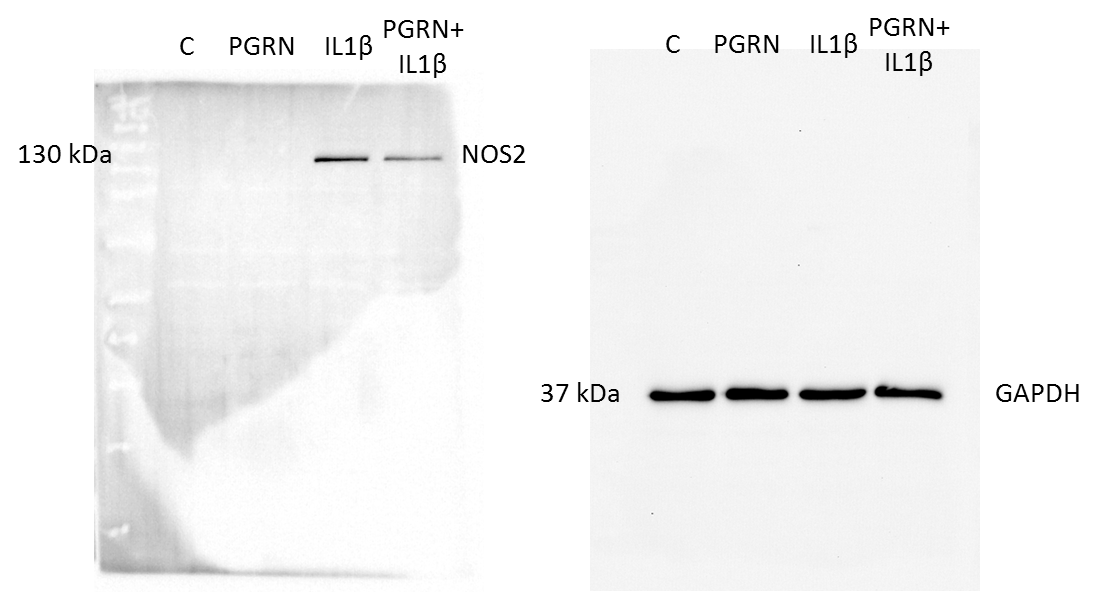
**


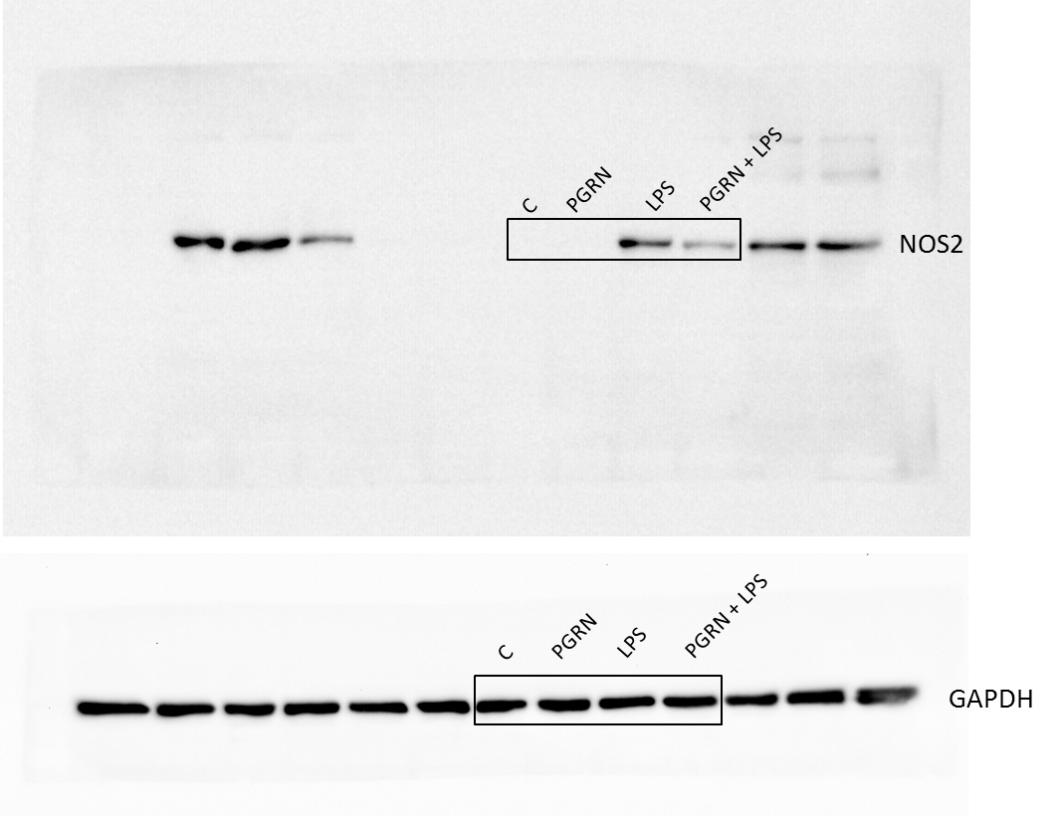

Supplement: Supplementary Information [file srep20356-s1.doc]
